# Bycatch of common pollinators in pheromone baited traps for monitoring corn earworm (Lepidoptera: Noctuidae) in Missouri Industrial Hemp

**DOI:** 10.1186/s42238-025-00266-y

**Published:** 2025-02-04

**Authors:** Clement Akotsen-Mensah, Isaac N. Ativor, Caroline N. Foba, Valliyodan Babu

**Affiliations:** 1 Cooperative Extension and Research, Lincoln University of Missouri, Jefferson City, MO 65101 USA; 2 Alabama Cooperative Extension System, Alabama A & M University, 4900 Meridian Street NW, Huntsville, AL 35672 USA; 3 Department of Natural Resources and the Environment, University of New Hampshire, Durham, NH 03824 USA

**Keywords:** Cannabis sativa, Helicoverpa zea, Non-target species, Pest, Pheromone-baited traps, bees

## Abstract

**Background:**

Several traps are recommended for monitoring corn earworm, *Helicoverpa zea* (Boddie), an important pest of field, vegetable and fruit crops in the U.S. These traps, which are meant to capture *H. zea* in many cases end up capturing other insects. *Helicoverpa zea* has recently been found feeding on different seeds and dual-type (seed and fiber) hemp, *Cannabis sativa* L. (Rosales: Cannabaceae) causing serious damage. Limited work has been done on developing integrated pest management (IPM) for *H. zea* industrial hemp in Missouri.

**Methods:**

We evaluated the attractiveness of different traps with the aim of developing a monitoring system for the adult male *H. zea* in industrial hemp fields in two Missouri locations. In addition, we recorded other non-target insects in the traps to determine trap selectivity. Commercially available green, clear, tricolor bucket traps Heliothis Scentry and Scentry Delta 1X traps baited with *H. zea* sex pheromones were evaluated in 2021 and 2022.

**Results:**

Tricolor traps captured significantly more adult male *H. zea* than the other traps in both years. Non-target insects, mainly *Xylocopa virginica* (L.) (Hymenoptera: Apidae), *Bombus* spp. (Hymenoptera: Apidae), and *Apis mellifera* L. (Hymenoptera: Apidae) were also captured. The tricolor trap captured the most bees.

**Conclusions:**

The presence of non-target species highlights the risk of using tricolor traps as a monitoring tool. This data provides information for planning the monitoring of corn earworm in industrial hemp farms in Missouri.

## Introduction

Production of industrial hemp, *C. sativa* has increased steadily in the U.S. since the introduction of the 2014 Farm Bill and the Agricultural Improvement Act of the 2018 Farm Bill [1, 2]. Hemp has been grown in the U.S. since the early 1600s, reaching peak production in the mid-1800s [3], with Missouri being second in hemp production in the U.S [4]. Hemp production was, however, restricted following the passage of the Marijuana Tax Act in 1937 [5]. As a result, between 1958, when the last hemp crop was planted in Wisconsin, and 1999, when a permit was issued for experimental test plots in Hawaii, no hemp was legally grown in the U.S. until the 2014 Farm Bill was amended [2].

Recognizing the potential benefits of hemp in the U.S. economy, Congress approved the legalization of hemp in the U.S. Currently, all U.S. states have federally legalized industrial hemp (herein referred to as “hemp”) production [1]. The value of hemp production in the open and under protection for the United States totaled $238 million in 2022 [6]. This includes food and body products, clothing, auto parts, building materials, and other products [7]. Since hemp has huge potential and has been approved for cultivation, it may become a field crop in the U.S., which may be grown in large acreages. As the production of this crop continues and expands, change in the arthropod complex and their abundance and damage is expected.

Like most field crops, hemp is infested by several arthropod species [8, 9], and the importance of these arthropod complexes may be significant as production of this crop continues to expand. Approximately 272 insects and mites are associated with *Cannabis* spp. globally [10]. In the U.S., about 150 insects and several arthropod pests are associated with hemp production and the pest management needs of some of the pests have been described [8, 9]. However, little to no information exists on the taxa and diversity of insects that attack hemp in Missouri. Based on our preliminary work in the 2020 hemp growing season, we found that corn earworm, *Helicoverpa zea* (Boddie, 1850), yellow-striped armyworm, *Spodoptera ornithogalli* (Guenée), brown stink bug, *Euschistus* spp. (Hemiptera: Pentatomidae), Southern green stink bug, *Nezara viridula* L. (Hemiptera: Pentatomidae), and green stink bug, *Chinavia halaris* (Say) (Hemiptera: Pentatomidae), and several other arthropods have established themselves as key pests of seed, dual (hybrid) and cannabinoid/floral varieties of hemp. Among these pests, *H. zea* [8], has demonstrated the greatest potential for crop injury in Missouri hemp. This report confirms previous observations by other researchers across several states [9, 11–13]. The larvae of *H. zea* cause significant damage to a diverse array of host plants, including some of the most cultivated crops like corn, tomato, pepper, soybean, etc [14]. *Helicoverpa zea* preferentially feeds on the floral regions of CBD and seeds of grain variety hemp [13].

Although, corn earworms, and the associated plants they infest, have been around for over a century [13], several studies have shown the population dynamics of this insect in many host plant plants except in industrial hemp. Limited knowledge exists on their monitoring except for work done using black light and various pheromone-baited traps mainly in corn (*Zea mays* L.) [15–17]. In addition, some studies have identified that the type of trap is critical when monitoring lepidopteran pests because of their potential to attract other non-targets like bees. One major trap design used in many monitoring studies is bucket traps with different colors and shapes. Also, the type of agricultural practices and landscape composition at various scales are known to shape the composition of bee and other arthropod communities [18–21]. There are many different sizes, shapes, and colors of traps. Designs have undergone extensive study, utilizing the unique characteristics of each insect that the trap is intended to catch. Certain insects respond to certain stimuli by moving in predictable ways, while others are drawn to particular colors or forms. Yellow is also a hue that attracts pollinators since it is found in flowering plants. Consequently, pollinators may inadvertently fall into yellow traps. In general, when moths are exposed to stimuli or use the moonlight to guide them, they fly upward. Consequently, moths are frequently captured using cone-shaped traps. In orchards, pests that damage growing fruits are monitored using red balls covered in pheromones and a sticky material [58].

Hemp is a prolific pollen producer, and most males produce an abundance of pollen that serves as vital subsistence resources to bees at a point in the season when they are resource-limited [22]. Hemp flowers attract a diverse range of bee species, and taller varieties of hemp attract a greater diversity of bees, making hemp a valuable resource for bees during floral scarcity and potentially benefitting other crops in the agricultural landscape [23]. Therefore, any pest monitoring in hemp should consider the effect that it will have on bee communities.

Since hemp is a new crop in Missouri, it is important to develop an IPM program that will permit better decision-making on how to suppress pest populations below the economic injury level. A critical step in developing an IPM program for any key pest depends on a good monitoring program that provides vital information on pests’ abundance and seasonality. Various methods have been used to monitor pest moths, including the use of pheromone-baited sticky traps [17, 24, 25], black light traps [26–29], drop cloths [30, 31] and pheromone-baited traps [17, 32, 33]. The performance of these traps in attracting corn earworms has not been well studied, particularly in hemp. In addition to these traps attracting lepidopteran pests, they have also been shown to attract other nontarget species (“bycatch”), such as bees. A good monitoring trap should, however, attract more of the target pest but few other nontargets.

In this study, we evaluated three trap types, namely the bucket, Heliothis Scentry, and Scentry Delta 1X traps that are frequently used to monitor several species of adult male moths in corn and other crops. These traps have produced different results in different agricultural production systems, particularly on bycatches of bees. Therefore, our objective was to evaluate and determine the effectiveness of some commercially available traps and sex pheromones for monitoring *H. zea* and their bycatch of bees in Missouri hemp fields. Ultimately, we aim to identify and recommend a good trap for effectively monitoring corn earworm adults while reducing bycatch of other nontarget insects in industrial hemp.

## Methods

### Study area and crop management

**Fig. 1:**
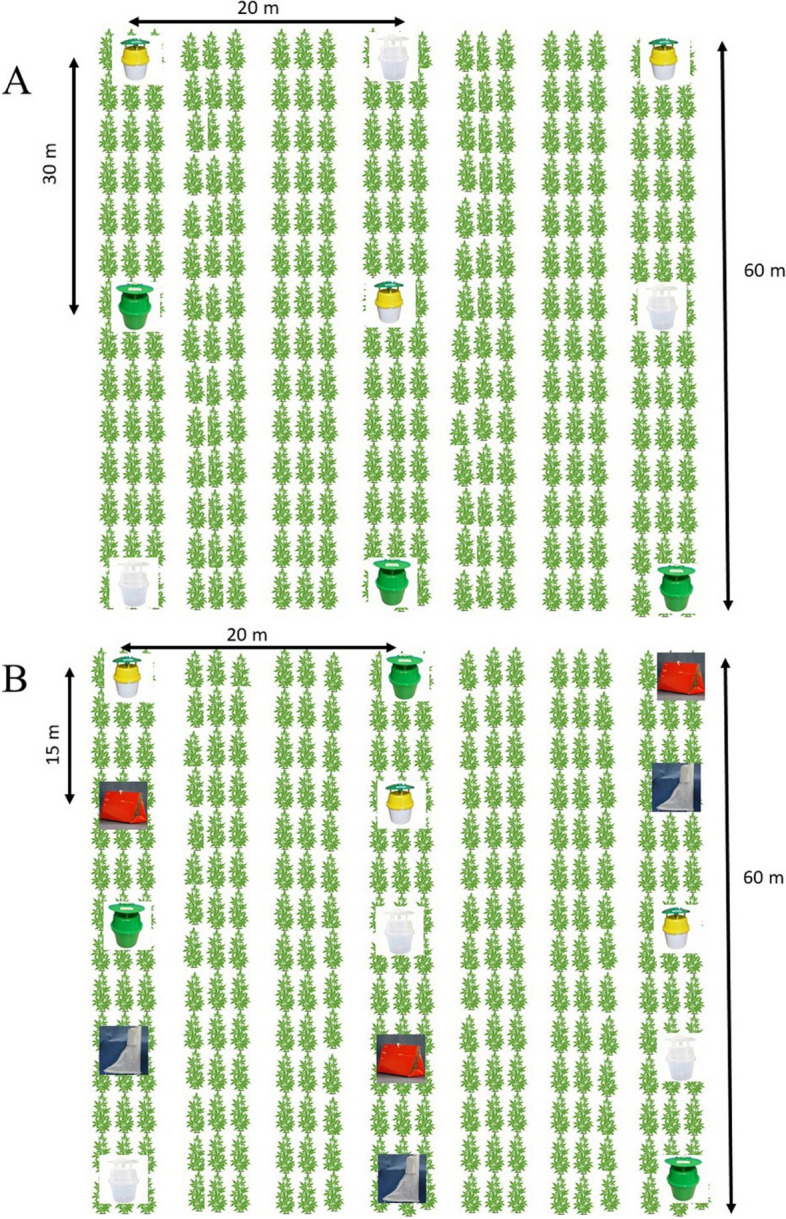
Layout of plots in **A**) 2021 and **B**) 2022. Traps were randomized in each row. A row was considered a block within which traps were randomized. In both years, each trap received the same corn earworm lure. Because the lures were the same, the maximum of 30 m (**A**) and minimum of 15 (**B**) distances between the traps in a row and between rows or blocks (20 m) were deemed adequate for insects to respond to the traps. This work did not test different lures but the type of trap.

The experiments were conducted in two Missouri locations, Lincoln University George Washington Carver, Jefferson City, MO (38^o^31’33.202”N 92^o^8’26.606”W) (herein referred to as Carver Farm), and Mid-West Natural Fiber AGRIPAK farm site located in Sikeston, MO (36^o^ 55’ 28.51”N 89^o^ 37’37.128”W) (herein referred to as Sikeston Farm) in 2021 and 2022 growing seasons (Fig. 1). These two locations represent the state’s Central and Southeast regions. Each field consisted of ~ 2 ha plots planted with different hemp varieties and types (dual, seed, and fiber). In both years, 32 varieties of 7 dual types, and 25 fiber types were planted in a variety trial. The dual varieties were planted at seeding rates of ~ 22.68 kg/ha and 18.14 kg/ha for the seed and fiber varieties, respectively. Seeds of all types and varieties were planted directly in the soil using a seeder mounted on a tractor. Seeding was done between May 15–22 each year and at each location. The plots were not managed in terms of weed control and pesticide applications. Agricultural weeds were abundant during the late seasons at all sites, which is more common in hemp than many other crops because no herbicides are registered for use on hemp in Missouri. The plots were irrigated when needed using overheard sprinkler irrigation systems.

### Description of experimental plots

At both locations, hemp seeds were planted on rows of 3 m by 30 m plots (Fig. 1A and B) and inter-row spacing of about 2.5 m at Carver Farm and 6 m at Sikeston Farm, depending on the tractor used.

### Sampling of moths using pheromone-baited trap

**Fig. 2:**
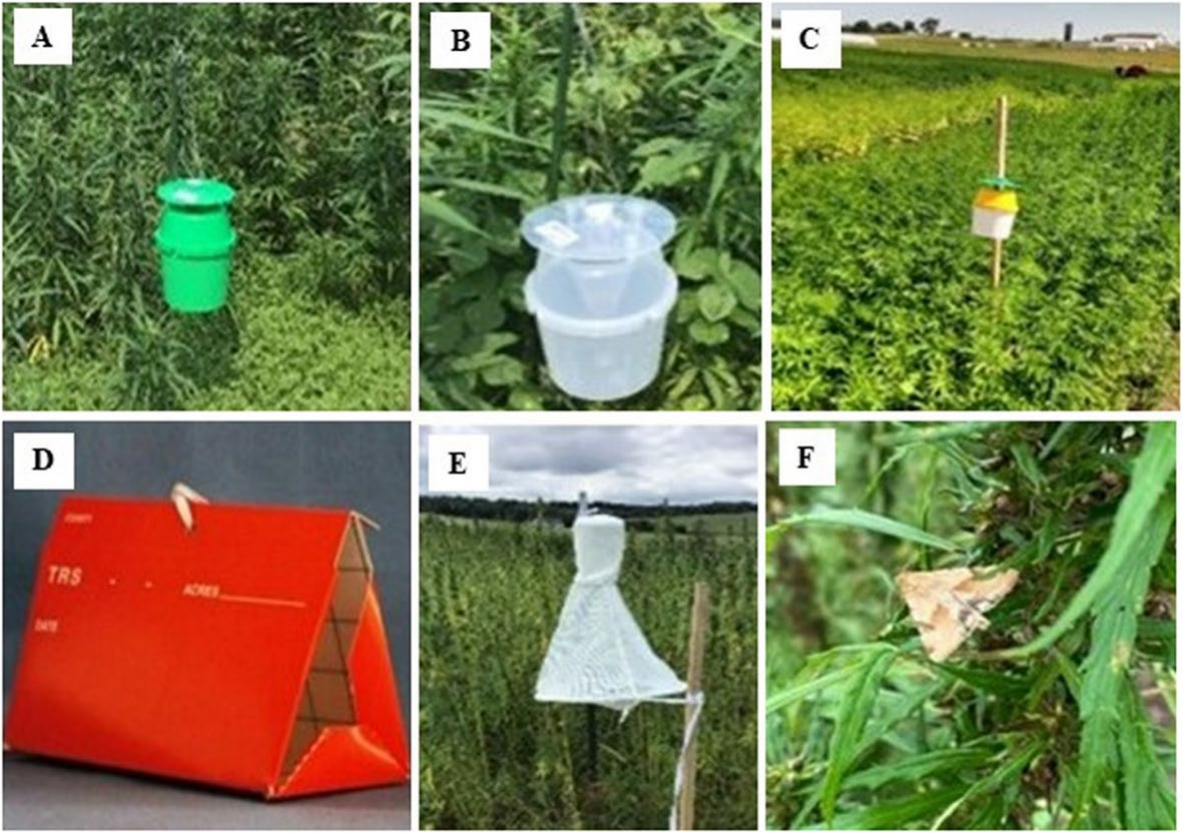
Bucket trap (green, clear and tricolor) (**A, B, **and** C**), Scentry Delta 1X (SD-1X) trap (**D**) and Heliothis Scentry (HS) (**E**). Picture of adult *H. zea* (**F**).

In 2021, three Trécé bucket traps of different colors (tricolor, green, and clear) baited with Trécé™ *H. zea* pheromone lure were evaluated to determine their performance in attracting male *H. zea* (Fig. 1-A). The traps and lures were purchased from Great Lakes IPM (Vestaburg, MI) and deployed in each site from August through September. The tricolor trap consisted of a white bucket (0.13 m tall and 0.16 m in diam.), a yellow funnel (bottom opening 0.03 m) on top of the bucket, and a dark green cover (0.16 m diam.) attached to the funnel by four posts that allowed a 0.03-m circular space in between cover and funnel (Fig. 2-A). The pheromone lure was placed in a green basket (0.05 m long) that hung in the middle of the green cover. The green (Fig. 2-A) and the clear (Fig. 2-B) bucket traps were similar in shape and size to the tricolor trap (Fig. 2-C), except that the entire unit consisted of green and clear colors, respectively. The pheromone lures for the green and clear bucket trap were placed in a green and clear basket-top cover. Traps were hung on 1.52 m wooden stakes using office clips and metal strings, so the trap height was about 1.45 m above ground. *Helicoverpa zea* rubber septa lures were replaced every six weeks. Traps within each block were rotated monthly.

The mechanism of moth capture was by direct flight into the bucket through the entrance in the trap top. Moths attracted to the pheromone “fell” downwards (usually after flying inside the posts and bouncing off the top cover) through the funnel into the bucket where they were killed through starvation. Traps within blocks and the separation among blocks were at least 15.00 m and 20.00 m apart, respectively. Traps were inspected weekly unless conditions did not permit. Adults of male *H. zea* from the traps were removed, held in Ziploc^®^ bags, counted, and recorded during each visit. Extreme care was taken to avoid the escape of live adults. Sampling was performed once every week. Although our focus was on *H. zea*, we also collected data on non-targets mainly bees (Hymenoptera: Apidae): Eastern carpenter bee *Xylocopa virginica* (L.), bumble bee *Bombus* spp., and honeybee *Apis mellifera* L. Each trap block was replicated three times and included one of each *H. zea* pheromone-baited bucket trap.

**Fig. 3:**
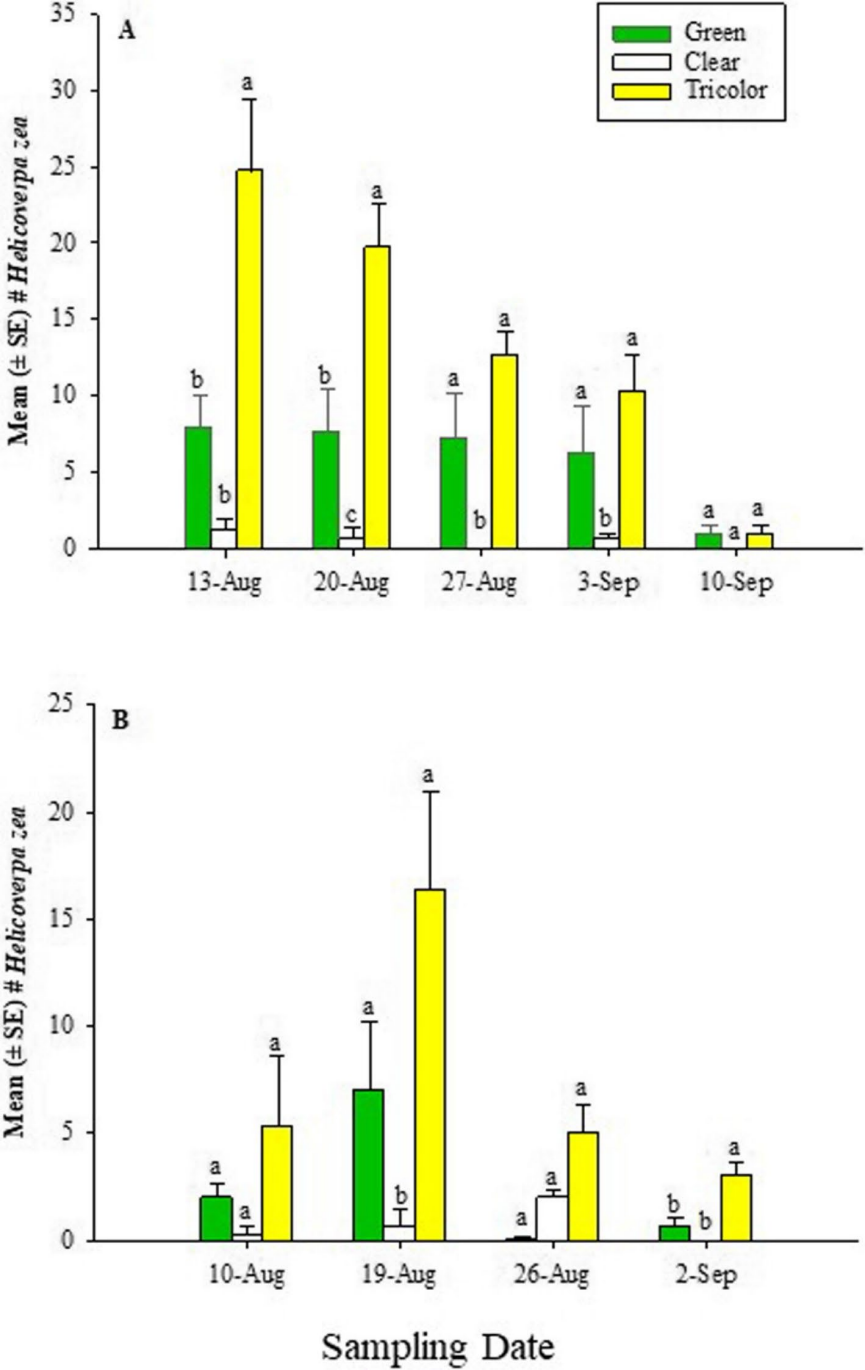
Mean (± SE) total number of adult male *H. zea* in traps at (**A**) Lincoln University Washington Carver Farm, Jefferson City, MO, and (**B**) Sikeston Farm during the 2021 hemp season. Different letters above bars indicate a significant difference (*P* > 0.05) among treatments within each sampling date. Mean separation was done using the Wilcoxon Chi-square approximation test.

In 2022, two additional traps, Heliothis Scentry [(HS), Fig. 2-E] and Scentry Delta 1X [(SD-1X), Fig. 2-D] traps, and Trécé™ were included except at the Sikeston farm where the HS trap was not included due to availability (Fig. 3).

For convenience, these traps are hereafter coded HS and SD-1X, respectively. Traps and lures were also purchased from Great Lakes IPM and deployed in each trapping site from May to September. The SD-1X trap measured 0.18 m in length by 0.10 m in width by 0.13 m height, with a sticky inner liner (0.17 m length by 0.10 m width by 0.09 m height). Moths captured in the SD-1X trap generally died overnight. Heliothis Scentry trap was composed of two cones. The base cone measured 0.80 m long, with a bottom opening of 0.34 m that narrowed to 0.15 m at the top; the apex cone measured 0.27 m long, with a bottom opening of 0.15 m that narrowed to 0.06 m at the top. The bottom portion of the apex cone was secured to the top portion of the base cone with Velcro material. The lure was placed along a cord stretching across the bottom of the base cone. Moths captured in the HS traps died over a couple of days. Traps were also hung on wooden stakes as previously described in 2021. *Helicoverpa zea* rubber septa lures were replaced every six weeks. Traps within each trap block were rotated monthly. Similarly, traps were replicated three times but included one of each *H. zea* pheromone-baited bucket traps (clear, green, and tricolor), HS, and SD-1X traps (Fig. 2). Traps within blocks and the separation among blocks were at least 15 m apart. Traps were inspected weekly unless conditions did not permit. Adults of male *H. zea* captured were removed from the traps, held in Ziploc^®^ bags, counted, destroyed, and removed from the field during each visit. Data on non-targets, mainly Eastern carpenter, bumble, and honeybees, were also recorded.

### Statistical analysis

Data for each year and location were first analyzed for ANOVA’s normality and heteroscedasticity assumptions. Data on *H. zea* larvae and adults that did not meet the assumptions of ANOVA were square root transformed. Where both actual and transformed data did not meet the assumptions of ANOVA, a generalized linear model was performed assuming Poisson distribution and log-link function to determine the effects of trap type, sampling date, and trap type * sampling date interaction. Where the trap type and sampling date interaction was significant, a one-way ANOVA was used to determine the performance of the trap types in each sampling date. A nonparametric Kruskal Wallis rank sum test was performed, and a Chi-square approximation (χ2) was used to determine the significant effect of trap type in each sampling date where applicable. All data were analyzed using JMP [34] and the results were considered statistically significant at *P* < 0.05. Non-metric multidimensional scaling (NMDS) ordination was used to visualize other hymenopteran and non-hymenopteran arthropod communities sampled in Lincoln farm in 2022. Data from Lincoln farm in 2022 was used because it supports multivariate analysis. Permutation-based nonparametric MANOVA calculated with the method of [60] (PerMANOVA) was used to test the comparison between the trap types. Analyses were based on Bray–Curtis distance.

## Results

### Trap performance in 2021

In 2021, at the Carver Farm, the GLM showed significant differences among sampling date (χ2 = 41.7, df = 4, *P* < 0.0001), trap type (χ2 = 86.8, df = 2, *P* < 0.0001), but the sampling date * trap type interaction was not significant (χ2 = 9.6, df = 2, *P* = 0.2901) for the adult *H. zea* captured in the traps. Analysis based on sampling date, showed significant differences on August 13 (χ2 = 7.3, df = 2; *P* = 0.0257), August 20 (χ2 = 7.3, df = 2, *P* = 0.0265), August 27 (χ2 = 6.7, df = 2, *P* = 0.0349), and September 3 (χ2 = 7.3, df = 2, *P* = 0.0496). However, no significant difference occurred in trap captures on September 10 (χ2 = 2.9, df = 2, *P* = 0.2301). In most of the sampling dates, tricolor traps captured significantly or numerically more *H. zea* than clear and green traps (Fig. 2a). At the Sikeston Farm, the GLM showed a significant difference for only the sampling date (χ2 = 33.4, df = 3, *P* < 0.0001), but trap type (χ2 = 3.5, df = 2, *P* = 1.0000) and the sampling date * trap type interaction (χ2 = 1.5, df = 6; *P* = 0.9586) were not significant. When the data were analyzed in terms of sampling date, the traps showed a marginally significant difference on August 19 (χ2 = 6.2, df = 2; *P* = 0.0457) and September 2 (χ2 = 6.0, df = 2, *P* = 0.0495) but not on August 10 (χ2 = 2.9, df = 2, *P* = 0.2301) and August 26 (χ2 = 0.00, df = 2; *P* = 1.0000) (Fig. 2b). The tricolor trap captured significantly and numerically more *H. zea* male adults compared to clear and green traps.

### Trap performance in 2022

**Fig. 4:**
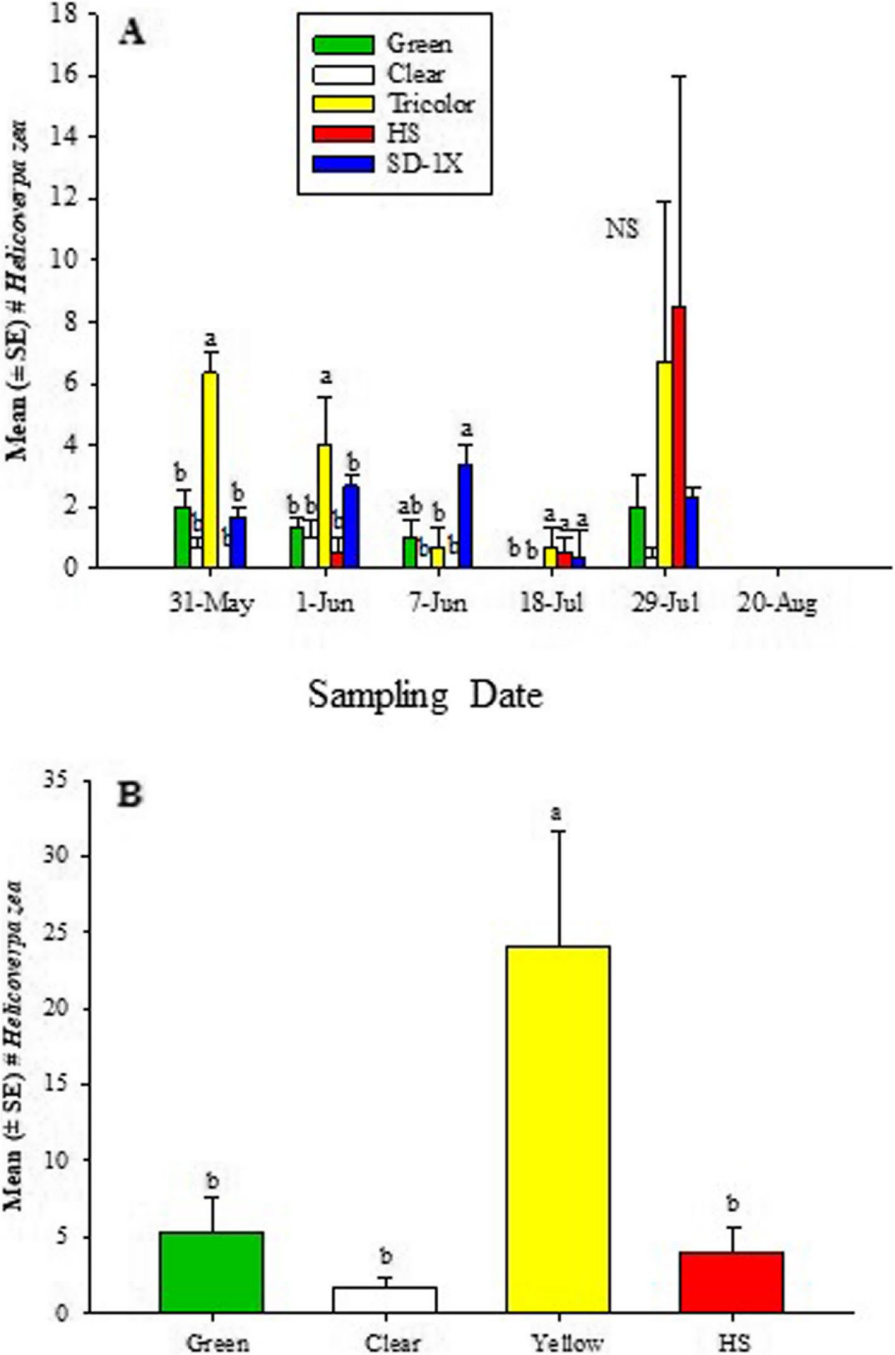
Mean (± SE) the total number of adult male *H. zea* traps at (**A**) Lincoln University Washington Carver Farm, Jefferson City, MO, and (**B**) Sikeston Farm, MO, during the 2022 hemp season. There was only one sampling date at the Sikeston location. Different letters above the bars indicate a significant difference (*P* > 0.05) among treatments within each sampling date. Mean separation was done using the Wilcoxon Chi-square approximation test.

In 2022, at the Carver Farm, the GLM showed significant differences among sampling dates (χ2 = 21.6, df = 4, *P* = 0.0002), trap type (χ2 = 48.1, df = 4, *P* < 0.0001), and the sampling date * trap type interaction (χ2 = 85.7, df = 16, *P* < 0.0001). When the data were analyzed in terms of sampling date, the traps differed significantly on May 31 (χ2 = 10.6, df = 4, *P* = 0.0309), June 1 (χ2 = 10.9, df = 4; *P* = 0.0281), June 7 (χ2 = 8.9, df = 4, *P* = 0.0542), July 18 (χ2 = 9.3, df = 4, *P* = 0.0548) and September 3 (χ2 = 7.3, df = 2; *P* = 0.0496). However, no significant difference was found on July 29 (χ2 = 4.3, df = 2, *P* = 0.3613) (Fig. 4a). In all the sampling dates, tricolor traps captured significantly or numerically more *H. zea* than green, clear, HS, and SD- 1X traps except on July 29 when HS recorded numerically higher *H. zea* males (Fig. 4a). At Sikeston, only one sampling date was recorded due to distance. The GLM showed a non-significant difference for only the trap type (χ2 = 7.6, df = 3, *P* = 0.0547) (Fig. 4b). Similarly, the tricolor trap captured significantly more *H. zea* than green, clear, or HS traps.

### Captures of common pollinators in 2021

**Fig. 5:**
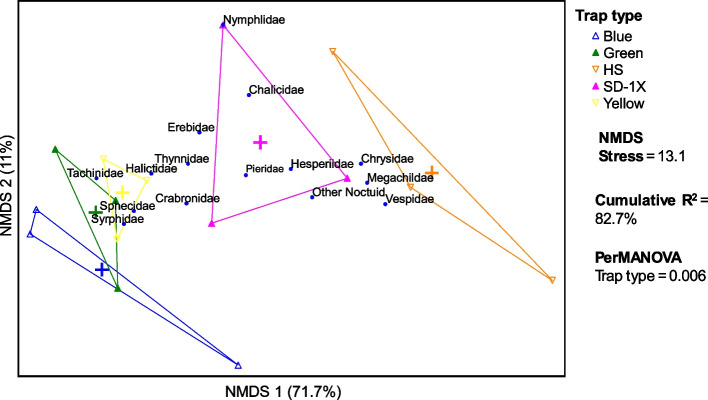
Nonmetric multidimensional scaling (NMDS) ordination of hymenopteran and non-hymenopteran arthropod communities sampled. Coefficient of variation (R^2^) Axis 1 = 71.7%, Axis 2 = 11%. NMDS Stress of 13.1. PerMANOVA, Trap type = 0.006.

In 2021, at the Carver Farm, a total of 204 bees were captured in the traps. Among the bees captured, 45.1% were carpenter bees, 28.3% were bumble bees and 26.5% were honeybees. The tricolor trap captured significantly more bees compared with clear traps, while no significant difference occurred between tricolor and green traps for all bee species (Table 1). At the Sikeston Farm, a total of 352 bees were captured in the traps. Among the bees captured, 19.6% were carpenters, 65.3% were bumble and 15.5% were honeybees. In both locations, the tricolor traps had significantly more bees compared with clear and green traps (Fig. 5; Table 1).

**Table 1:** Mean (± SE) number of common bee species captured per sampling week in traps in 2021

Location	Trap type	Carpenter bee Mean ± SE	Bumble bee Mean ± SE	Honey bee Mean ± SE
Carver Farm	Green	1.40 ± 0.53 ab	1.27 ± 0.51 a	1.07 ± 0.73 a
	Clear	0.47 ± 0.24 b	0.33 ± 0.19 a	0.60 ± 0.47 a
	Tricolor	4.27 ± 1.39 a	2.27 ± 0.88 a	1.93 ± 1.47 a
	*χ*^*2*^	*9.12*	*5.38*	*0.33*
	*df*	*2*,* 44*	*2*,* 44*	*2*,* 44*
	*p*	*0.0104*	*0.0679*	*0.8473*
Sikeston	Green	0.58 ± 0.26 b	1.33 ± 0.61 b	1.33 ± 0.45 a
	Clear	1.08 ± 0.34 b	2.92 ± 0.94 b	1.25 ± 0.49 a
	Tricolor	4.08 ± 1.08 a	14.92 ± 4.67 a	1.83 ± 0.58 a
	*χ*^*2*^	*9.15*	*9.75*	*0.69*
	*df*	*2*,* 33*	*2*,* 33*	*2*,* 33*
	*p*	*0.0103*	*0.0076*	*0.7091*

Means followed by different letters in the same column are significantly different at *p* = 0.05. Means followed by the same letter within each column are not significantly different (*P* > 0.05, Chi-square approximation)

### Captures of common pollinators in 2022

In 2022, at the Carver Farm, the traps baited with *H. zea* lures captured a total of 553 (*N* = 75) bees. Among the bees captured, 15.9% were carpenter bees, 66.0% were bumble bees and 17.2% were honeybees. At the Sikeston Farm where only one sampling date was performed, the traps that contained *H. zea*, captured a total of 117 bees (*N* = 12). Among the bees captured, 15.9% were carpenter bees, 66.0% were bumble bees and 17.2% were honeybees. In both locations, the tricolor trap had significantly and numerically more bees than green, clear, HS and SD-1X traps (Fig. 4; Table 2). Ordination with NMDS indicated that other hymenopteran and non-hymenopteran arthropod communities were relatively heterogeneous/differed by the trap types (trap type PerMANOVA = 0.006) (Fig. 5).

**Table 2:** Mean total (± SE) number of bees captured in traps baited with *H. zea* lures in two locations in 2022

Trap type	H. zea	
	Carver farm	Sikeston farm
Green	11.00 ± 2.08 b	10.67 ± 2.33 b
Clear	8.3 ± 2.70 b	2.67 ± 0.89 b
Tricolor	134.6 ± 56.2 a	31.67 ± 1.8 a
HS	14.0 ± 2.73 b	NA
SD-1X	16.33 ± 4.9 b	22.00 ± 6.5 ab
*χ* ^*2*^	*8.8*	*8.00*
*df*	*4*	*3*
*P*	*0.064*	*0.0459*

Means followed by the same letter within each column are not significantly different (*P* > 0.05, Chi-square approximation). HS is the Heliothis Scentry trap and SD-1X is Scentry Delta trap

## Discussion

In this study, we sought to determine the capture of different traps for corn earworm monitoring and identify the by-catch of the bee community captured in the corn earworm pheromone-baited traps in hemp. In 2021, we found significant differences in captures among the different bucket traps with the tricolor bucket trap recording significantly more adult *H. zea* males than green and clear bucket traps in most of the sampling dates. In 2022, the tricolor trap was still better at capturing more *H. zea* even when the standard Scentry Heliothis (HS) and Scentry Delta (SD-1X) traps were added. Even though we did not include the homemade plastic yellow jug trap, which was previously reported to be more attractive than other trap colors [38, 41], we presumed the presence of the yellow and white colors in the tricolor trap synergistically contributed to the high captures of the adult *H. zea.* Because the same pheromone dispensers, pheromone load, release rates, lures longevity, and trap height were used, we believe these factors contributed equally to the attraction of *H. zea* adults into the traps.

Insects use olfactory and visual stimuli to locate resources that have been used to monitor many insects successfully [35, 36], particularly moths, flies, and beetles. We consider that both visual stimuli provided by the tricolor trap and olfactory stimuli provided by the *H. zea* pheromone contributed to *H. zea* being attracted to the traps, even though a standard yellow trap was not included in the trapping design. In contrast, previous studies have also shown that homemade yellow traps have been the most effective in attracting and capturing various insect species, including *S. frugiperda* [60–62].

Although both olfactory and visual stimuli acted to attract *H. zea*, color played a major role in the performance of the traps since there was a significant difference between the traps with the same design but different colors and those with different designs. The response of insects to color varies, but color can enhance trap numbers in many situations. In a similar study that evaluated different traps in capturing wild male velvet bean caterpillar moths (VBC), *Anticarsia gemmatalis* Hübner (Lepidoptera: Erebidae), and fall armyworm moths (FAW), *Spodoptera frugiperda* (J.E. Smith) (Lepidoptera: Noctuidae), significantly fewer VBC and FAW males were captured in pheromone-baited mono-colored (forest green) bucket traps than standard multicolored bucket traps [37]. Our inability to measure the reflectance and hue of the different trap colors used in this study is a major setback. However, several authors have shown that the compound eyes of some noctuid moths have a bimodal sensitivity to light with peaks in the UV (350–370 nm) and green (500–575 nm) regions [37, 38]. Yet, other noctuid species respond more strongly to traps emitting low spectral reflectance in these regions [39, 40]. Although green and tricolor are ubiquitous colors in the landscape, it is reasonable to expect that combining these colors will enhance trap capture. However, studies show that not all of these colors can enhance trap captures [41].

The capture of different bee species in the traps was not surprising because several studies investigating pheromone traps for moth monitoring resulted in the capture of non-targets including bees [42–44]. Due to its temporally unique floral phenology, hemp has the potential to provide an important food resource to diverse bee communities during periods of flower scarcity, thereby providing support for other crops in landscapes across agroecosystems [23].

The main non-target species found in the traps were carpenter, bumble, and honeybees. These were particularly high in numbers in the tricolor bucket trap, contrary to the report that bees usually respond to blue and yellow colors, but more to blue color. Studies on a different project using blue vane traps support the hypothesis that bees respond to blue color [Akotsen-Mensah unpublished]. In 2021 and 2022, a total of 757 bees were captured in the traps. Bycatch of non-target insects in monitoring traps has been shown to increase trap processing time, decrease trap effectiveness for the target species, and can include beneficial arthropods that provide important ecosystem services [42, 43]. Beneficial species frequently captured in monitoring traps might negatively impact their populations and the services they provide. The trap type with clear (i.e., no color) trap collected the least trapped beneficial insects while the tricolor trap attracted the highest populations. This means the lures play a pivotal role in attracting non-target insects. In a study by [59], commercially available lures attract fewer non-target insects compared to traps baited with the other lures and the trap color did not have a significant effect on the capture of *S. frugiperda* or *A. ipsilon* males [59].

Hymenopteran pollinators comprise much of the insect bycatch in moth pheromone-baited traps in various agroecosystems [45–49]. Although the impact of bee removal from agroecosystems in monitoring traps that target moth pests is unknown [46], it may negatively influence local bee abundance and species diversity, which could alter pollination services for both wild plants and managed crops and could reduce crop yields [50, 51]. As there is already widespread recognition of global declines of wild bee populations [50–52], practices that systematically remove bees from agricultural landscapes should be avoided. Although the tricolor traps did significantly better than green and clear traps, their ability to also attract bees may not make this trap a good monitoring tool. The reason is that four out of five North American bumble bee species, one of which was captured in our study have been earmarked on the list of Endangered Species Act [53]. Since pollinator declines can have cascading effects on associated ecosystems [54–56], any trap that will attract pollinators may not be accepted by growers [57, 58, 39].

If the tricolor bucket trap should be used to monitor *H. zea* in the hemp field because it captured more of the targeted species (i.e., *H. zea*), it will be appropriate to find a way to reduce its ability to capture bees and other pollinators. At this point, we can recommend the green bucket trap as an alternative since it was also able to attract *H. zea* but had fewer bees. The high number of bees in the field may be due to the search for pollen from the male hemp plant, which was in abundance in the field. Since the plot we used was a variety trial with many male flowers that shed pollen, it is possible many bees came to the field in search of pollen and eventually ended up being captured by the traps based on the colors.

## Conclusions

This study has confirmed that *H. zea* (both adults and larvae) can be present in industrial hemp fields in Missouri from the time of planting (usually mid-May) until harvest (end-September). The presence of these insects may pose significant threats to hemp seed production. Tricolor bucket traps should be installed early in the season to detect these insects. However, due to concern about the tricolor trap capturing bees, particularly carpenter and bumble bees, this trap can be substituted with the green color bucket trap, if the monitoring goal is to detect the insects. Our results have provided information on monitoring these insects, particularly *H. zea*. Although our research aims to use the trap numbers to make management decisions, it is too early to use the trap numbers to guide insecticide applications. We suggest further studies to determine the economic threshold of hymenopteran and non-hymenopteran arthropod communities, insect damage and landscape and weather factors to better understand the population dynamics and economic damage of these insects. The information obtained can be useful for effective insecticide applications for production profitability and environmental sustainability of industrial hemp in Missouri.

## Data Availability

No datasets were generated or analysed during the current study.
